# Deprescribing: Practical Ways to Support Person-Centred, Evidence-Based Deprescribing

**DOI:** 10.3390/pharmacy7030129

**Published:** 2019-09-03

**Authors:** Katherine Le Bosquet, Nina Barnett, John Minshull

**Affiliations:** 1 Guy’s and St Thomas’ NHS Foundation Trust, London SE1 7EH, UK; 2 NHS Specialist Pharmacy Service, London North West University Healthcare NHS Trust, London HA1 3UJ, UK

**Keywords:** deprescribing, shared decision making, person-centred, medicines optimisation

## Abstract

Deprescribing is complex and multifactorial with multiple approaches described in the literature. Internationally, there are guidelines and tools available to aid clinicians and patients to identify and safely withdraw inappropriate medications, post a shared decision-making medicines optimisation review. The increase in available treatments and use of single disease model guidelines have led to a healthcare system geared towards prescribing, with deprescribing often seen as a separate activity. Deprescribing should be seen as part of prescribing, and is a key element in ensuring patients remain on the most appropriate medications at the correct doses for them. Due to the complex nature of polypharmacy, every patient experience and relationship with medications is unique. The individual’s history must be incorporated into a patient-centred medication review, in order for medicines to remain optimal through changes in circumstance and health. Knowledge of the law and appropriate recording is important to ensure consent is adequately gained and recorded in line with processes followed when initiating a medication. In recent years, with the increase in interested clinicians globally, a number of prominent networks have grown, creating crucial links for both research and sharing of good practice.

## 1. What is Deprescribing?

Deprescribing is the process of intentionally stopping a medication or reducing its dose to improve the person’s health or reduce the risk of adverse side effects [1]. Deprescribing is a hot topic, with international interest driving new policy across the globe. Within this article, we will discuss why deprescribing is important and must involve shared decision making. We will reference the importance of documentation and the law, and tools and networks that can be utilised to ensure deprescribing is safe, effective, and patient inclusive, with the aim of improving quality of life.

Deprescribing is a well-documented challenge due to the multifactorial nature of polypharmacy and the patient experience. A systematic review conducted to ascertain the key elements of polypharmacy prescribing appropriateness indicators identified 20,879 abstracts, with 22 papers reviewed for information on indicators. The authors identified 12 polypharmacy indicators that could be used in clinical practice [2]. The research shows the complexity of deprescribing, with papers reviewing different interventions, ranging from audits to identify poor prescribing practice and intervention to individual case reports for the deprescribing of complex medications for the frail multimorbid polypharmacy patient [3]. Just as with initiating a medicine, deprescribing is unique in every situation, as people on identical medicines with the same co-morbidities will be influenced by different genetics and experiences.

**Figure 1:**
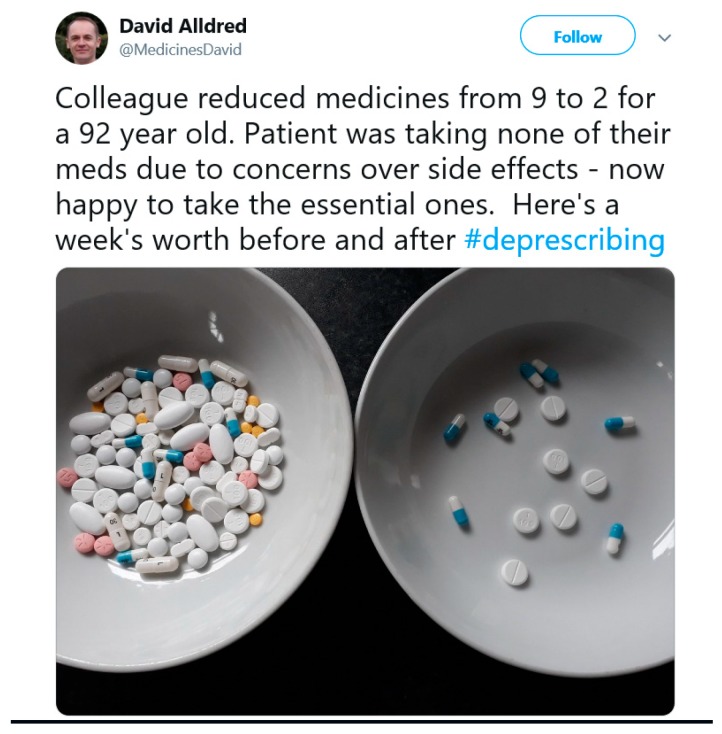
David Alldred’s viral deprescribing tweet [4].

This challenge is not just evident in literature, but also on social media; a picture tweeted (Figure 1) following a medication review with a 92-year-old patient concerned about the side effects of their medications generated 270,000 impressions and 1100 retweets across the world [4]. This tweet provides a visual impression of the change in the pill burden and the high level of inappropriate polypharmacy for this patient. This sparked conversation in the clinical community not for its extreme nature, but because this is the reality for many patients seen today.

While advances in medicines have increased life expectancy and quality of life for many with long-term conditions, there is a growing need to ensure patients remain on the best medication for them, considering personal experience and circumstance, with no one size fits all. Medication optimisation reviews are required to strike a balance between risk and benefit for every patient.

## 2. Inappropriate Polypharmacy, Medicines-Related Harm, Medication Review

There is increasing concern in the public that the healthcare system is overdosing, overtreating, or over diagnosing patients and that over diagnosis and treatment poses a threat to human health [5]. At the same time, there is concern about rationing and restricting treatments [6].

The risk: benefit balance changes throughout a patient’s life due to additional co-morbidities, changing circumstances, additional prescribed medications, or the physiological changes that come with ageing [7]. This highlights the need to clearly document the indication for a medication on initiation. This is because stopping a medication is much more difficult when the indication is not clear.

**Figure 2:**
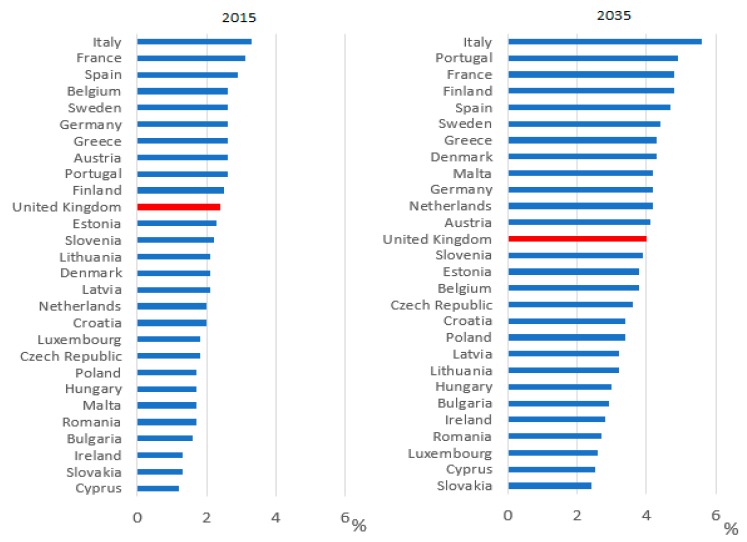
Percentage of population aged 85 and over in EU countries in 2015 and 2035 (projected) [10].

Systems within healthcare are set up to facilitate prescribing and initiating new medicines when they are indicated. Yet the systems are not as robust to support stopping medicines when they are no longer indicated or are causing more harm than benefit. The number of medications prescribed per patient is resultantly gradually increasing [8]. The availability of new products and patients living longer with long-term conditions means this trend is likely to continue unless there is a change in practice around prescribing. With adverse drug reactions accounting for more morbidity and mortality than most chronic diseases [8], this increase in prescribing needs to be addressed. In England, the NHS Business Services Authority (BSA) prescribing data shows that in December 2018, for patients who take at least one medication, 5.22% were prescribed 10 or more unique medicines, rising to 10.27% for patients aged 85 and over [9]. In 2016, from a population of 65.7 million, over 18% of the population (11.8 million and rising) were over 65 and 1.6 million people (2.4% of total population) were aged 85 and over [10]. Internationally there is a projected increase in the number of older people, as shown in Figure 2 [10].

Prior NHS England GP contracts required practices to identify frail patients and ensure patients with severe frailty receive an annual medication review, however did not specify the level of review expected, nor who should carry this out. The new GP contract five-year framework (2019/20–23/24) delivers new services to meet the NHS long-term plan commitments, including the creation of primary care networks; within the contract is provision of “structured medication review” due to be started by April 2020 [11].

The World Health Organization (WHO) have identified harm from medication as a priority and are completing the third global safety challenge: Medication without harm. The challenge aims to reduce events of severe avoidable harm in relation to medication by 50% over five years, globally, with polypharmacy included as one of three key areas for action [12].

England approached the WHO challenge by setting up the Medicines Safety Programme. This programme commissioned a report into medication harm, which found that in the UK alone, there are an estimated 237 million medication errors occurring annually, with 68.3 million errors (28% of total) causing moderate or serious harm. The estimated NHS costs of definitely avoidable adverse drug reactions is £98.5 million per year, consuming 181,626 bed-days, causing 712 deaths, and contributing to 1708 deaths [13].

Medication errors are more likely to occur in older people, or in the presence of co-morbidity and polypharmacy [8]. For older adults in the UK, a study into medication within eight weeks of discharge from hospital found 37% of patients experienced harm from their medication. Serious harm accounted for 81% of cases with 52% potentially preventable [14]. 

## 3. Risks and Benefits of Deprescribing

There are two distinct overarching principles to reducing the harm from inappropriate polypharmacy identified above:

- Changing the culture around prescribing to reduce unnecessary medication being started.
- Deprescribing medications that are no longer beneficial for the patient.

**Figure 3:**
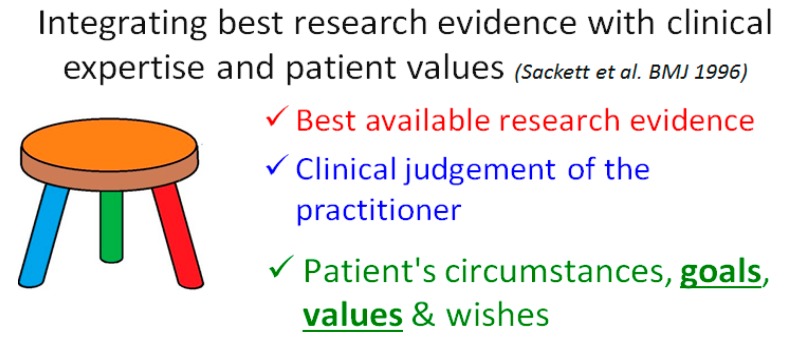
Integrating best research evidence with clinical expertise and patient values [15,16].

To reduce this risk of harm, all patients need medication reviews to identify the most effective and safe medications—considering evidence-based medicine, the patient’s experience, and full medical history. Identified medications that do not meet these criteria should be discussed with the patient and consideration given to deprescribing. Figure 3 shows the three key elements of medicines optimisation, originally described by Sackett et al. in 1996 in relation to evidence-based medicine [15].

Deprescribing medications must be viewed by the prescriber in the same way as prescribing or continuing a medication, with the potential for harm as well as benefit [17]. Potential for—or fear of—harm when stopping a medication is often cited as a barrier to deprescribing, alongside lack of time, differing motivations of the prescriber, lack of knowledge, or uncertainty and communication. These barriers appear in international literature, including papers from Singapore, the USA, and the UK [17,18,19].

Further investigation into the barriers to deprescribing identified four main safety concerns/potential harms of deprescribing in older adults:

- Adverse drug withdrawal events;
- Return of medical condition(s);
- (Unpredictable) reversal of drug-drug interactions; and
- Damage to the doctor–patient relationship [17].

It is key to recognise these barriers with clinicians and empower them to manage deprescribing appropriately, given that there is an unknown outcome when stopping any medication, be this a benefit or a harm. It is important to note that continuing a medication that has no current indication and that has a potential to cause medication-related harm is not in the best interests of the patient. Prescribers who continue to prescribe a medication without providing the benefits and options for deprescribing could be exposed to clinical negligence cases, and would not meet the full legal requirements for informed consent as described by Barnett and Kelly [20].

In addition, deprescribing is desirable as a collaborative process: A study found in Australia 88% [21], America 66.6% [22], and Malaysia 67.7% [23] of older adults would like to reduce or try stopping medicines when recommended by a doctor. Patients are keen to be involved in the process of deprescribing, with a study finding 78.9% of the elderly would like to participate in the deprescribing process [24].

## 4. Deprescribing and the Law

While law relating to deprescribing is limited, it is viewed as any other clinical activity and thus fits within existing legal frameworks. Clinicians should consider stopping a medicine in the same way as they would consider the act of initiating a medication—treating it with the same importance and care. However, while there is a plethora of guidance to support safe initiation of medication, the evidence base for discontinuation is, at best, variable. Current clinical guidelines are often based on evidence proven in younger/healthier adult populations using a single disease model [25]. This evidence base decreases as co-morbidities and medications increase and decreases further with age, with older patients rarely included in randomised controlled trials. As single condition guidelines for prescribing rarely contain guidance on stopping a medication, prescribers can be left feeling unsupported and may fear the risk of litigation if something were to happen to the patient [20].

Additionally, many prescribers view deprescribing as a separate activity focusing on a specific subset of patients: Those on polypharmacy, living with frailty, initiating deprescribing clinics with a focus on in-depth review of medications. However, deprescribing should be part of prescribing [9]: Whenever a medication is prescribed, there should be a review of the current medications with the patient, and any medications that are not effective, reduced or stopped.

## 5. Shared Decision Making, Informed Consent, and Deprescribing

NICE and NHSE created a consensus statement for shared decision making (SDM) in 2016, stating that “SDM is a process in which clinicians and patients work together to select tests, treatments, management or support packages, based on evidence and the patient’s informed preferences. It involves the provision of evidence-based information about options, outcomes and uncertainties, together with decision support counselling and a system for recording and implementing patients’ informed preferences” [26].

SDM is key to keeping within the legal framework in the UK for consent when deprescribing and preventing any clinical negligence cases from arising—it applies equally to deprescribing and prescribing, and literature around prescribing and SDM is applicable to deprescribing. In the UK, the principles of clinical consent are informed by the Montgomery judgement [27], which states that patients must be informed of risks considering the needs, concerns, and circumstances of the patient when deciding about starting or stopping a medication [28]. Similarly, a high court ruling in Australia, Rogers vs. Whitaker [29,30], stated the clinician has “a duty to take reasonable care to ensure that the patient is aware of any material risks involved in any recommended medical treatment, and of any reasonable alternative or variant treatments” [27].

Some patients may not wish to be involved in the discussions regarding treatment and it is the clinician’s responsibility to assess the amount of involvement the patient wants. The clinician can then provide sufficient information to the patient about why one treatment may be medically preferable to another, making the patient aware of all options, with the advantages and disadvantages of each [28]. 

## 6. Person-Centred Deprescribing, Medicines Adherence, and “Self-Deprescribing”

It is estimated that 50% of patients do not take their medication as prescribed [31]. Patients make choices about whether to take their medicines or not, despite—or because of—what they have been told by health professionals. An open conversation about medicines adherence can allow clinicians to work with the patient to mitigate their concerns.

When deprescribing, it is important to understand the patient perspective on stopping medicines. Patients often have strong views on which medications to stop or continue, which must be considered in the medication review process. Patients who feel the process is happening to them, not with them, will often disengage in the review. Focusing on a problem that is unimportant to the patient whilst ignoring their main concern is also unlikely to be productive—i.e., attempting to adjust blood pressure tablets in a constipated patient or change the inhalers for a patient in pain. Behavioural scientists have identified multiple individual reasons for non-adherence for patients across capability, opportunity, and motivations, and that the reasoning behind non-adherence for each medication can be different [32].

**Figure 4:**
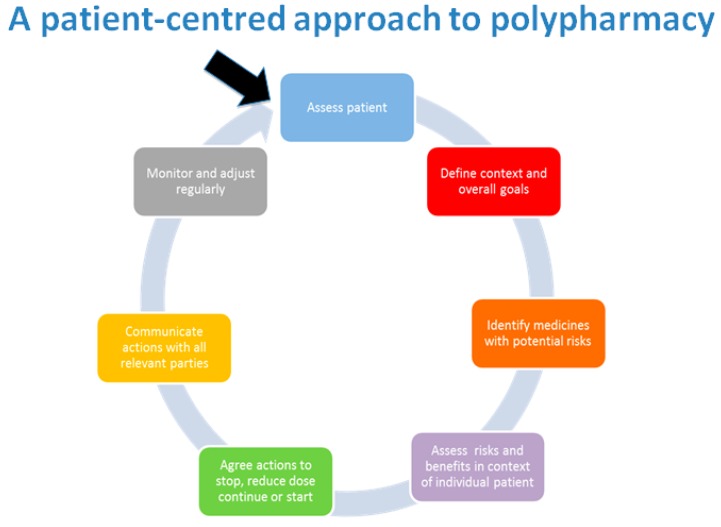
Patient-centred management of polypharmacy a process for practice [34].

From experience, the authors have found that when a patient fully engages in discussions, it is easier to identify non-adherence and adverse drug reactions and side effects attributable to one or more medications. Clarifying the side effect history can aid your review by helping identify where a patient might be caught in a prescribing cascade, where new medications are added to treat side effects of previously prescribed medications [33]. The patient-centred approach to polypharmacy is a step-wise approach to medication review, as show in Figure 4. This method assists clinicians in ensuring reviews are completed in a structured manner. This can be used alongside the tools featured later in this article.

Where adherence issues are not discussed, patients may choose to “self-deprescribe” by stopping a medication without discussing it with their prescriber. Health psychologists working in this area suggest that there are a variety of reasons to “self-deprescribe”, including side effects, lack of obvious efficacy, or difficulty in accessing the product. This must be completed whilst recognising that patients can have more than one reason for stopping a medication, or different reasons for each medication [33]. This highlights the importance of open, trusting conversations between prescriber and patient when discussing stopping or continuing a medication. There is also an important safety issue when people are admitted to hospital. If hospital clinicians believe that patients are taking all prescribed medicines when they are not, all prescribed medications will be administered, exposing the patient to medications they have chosen to stop.

## 7. International Deprescribing Tools

Deprescribing and support aids have been developed internationally in response to the world-wide desire to approach deprescribing in a structured manner. Tools to assist with identifying medications that may be causing harm or that need a review may support the deprescribing process. There are multiple tools to support deprescribing. One systematic review (citing papers up to December 2017) identifying 15 Tools for use during the process of deprescribing in frail older persons and those with limited life expectancy [35]. Most of the tools identified were developed utilising expert opinion and ranged from complex algorithms to simple lists or flow charts to follow in consultation. Some had poor description of development methodology and only a few tools have been tested in clinical trials, with only four of the studies identified in this review having been tested (low-quality evidence) [35].

Tools to improve prescribing and aid clinical decision making include both “explicit” and “implicit” approaches to medication review. “Explicit” tools list the medications to review by name, whilst “implicit” tools suggest questions a clinician may wish to ask to identify whether a medicine is still needed or appropriate [36]. 

Explicit tools include:

- Australia—NSW Therapeutic Advisory Group Deprescribing guides [37].
- Canada—Deprescribing guidelines and algorithms [38] and MedStopper [39].
- Ireland—STOPP/START [40] and STOPPFrail [41].
- England—Anticholinergic Burden (ACB) Risk Scales [42].
- Italy—CRIME (CRIteria to assess appropriate Medication use among complex Elderly patients) [43].
- USA—Beers Criteria Update 2019 [44].
- Germany—FORTA (Fit fOR the Aged) [45].
- Germany—PRISCUS [46].

Implicit tools include:

- MAI—Medication Appropriateness Index [47].
- Scotland—7 Steps and Polypharmacy App [48].
- Wales—Polypharmacy guidance—flow diagram for deprescribing [49] and NO TEARS [50].
- Israel—The Good Palliative-Geriatric Practice (Garfinkel) Algorithm [51].

## 8. Deprescribing Networks

Interested clinicians are combining their expertise to establish networks across the globe, using social media and the production of guidelines and tools to continue this culture shift within prescribing to include deprescribing at the point of prescribing routinely. This culture change will need to permeate through every system and stage of healthcare, including educating the general population about the right to be involved in the decisions surrounding their medications and care, increasing patient activation levels, and ensuring medications are adequately reviewed and stopped in all sectors when the benefits outweigh the harms for the patient.

The Canadian Deprescribing Network (CaDeN) and the Australian Deprescribing Network (ADeN) are leading the way for sharing good practice through collaboration of interested parties, and are creating guidelines and algorithms to assist clinicians in the technical removal of a medication safely with appropriate follow-up. These groups are also assisting in the creation of policy to drive forward the ambition nationally; an example of this is ADeN’s work on Australia’s Quality Use of Medicines to Optimise Ageing in Older Australians [52].

These tools add to the variety of tools currently available internationally, often providing reassurance and a logical step-wise process for deprescribing for clinicians and patients to follow, but in the UK, these are not written into clinical guidelines—i.e., NICE guidance as for prescribing. These tools are a key support for clinicians in safe deprescribing, as instructions on the monitoring and methods for deprescribing are rarely contained within the manufacturer’s recommendations or British National Formulary, except in the case of medications with documented withdrawal effects.

The deprescribing.org guidelines are robust guidelines, and algorithms created using a defined systematic review process are documented and provided openly; they provide practical recommendations for making decisions about when and how to reduce the dose of or stop medication. Recommendations are meant to assist with, not dictate, decision making in conjunction with patients [38].

Following the success of CaDeN and ADeN, Europe has followed suit with the recently launched English Deprescribing Network (EDeN) and the more academic and researcher-focused Northern European Deprescribing Network (NERD) and International Group for Reducing Inappropriate Medication Use & Polypharmacy (IGRIMUP), who have presented research into deprescribing at conferences and have written a position statement for future works [25]. 

## 9. Recommendations

Reading the literature, and from the authors’ experiences, we recommend:

1. Deprescribing should be seen as part of the prescribing process and:
  - Should occur as part of a shared decision -making process, focusing on the patient’s main problems.
  - Working with the patient to find solutions is likely to support this relationship.
  - The prescriber–patient relationship can be put at risk if the deprescribing conversation is not sensitively undertaken.
2. Medication should be stopped when the patient or carer and clinician decide together that the medication’s benefits no longer outweigh the risks for that patient, including:
  - Use of tools is advisable to aid in the identification of medications that require review and to provide advice for the safe removal or reduction of medication.
  - Ensure patients are involved in creating the full plan for review and encourage them to report adverse effects when stopping or reducing medication to develop and maintain effective, trusting relationships around deprescribing.
  - Clear documentation is important to ensure meeting legal requirements and, as with prescribing, consent is key.
3. It can be helpful to find networks in your area or join an international network for the quickest access to new tools, advice, and evidence or to share experience.
